# Spatio-Temporal Distribution of Dengue and Lymphatic Filariasis Vectors along an Altitudinal Transect in Central Nepal

**DOI:** 10.1371/journal.pntd.0003035

**Published:** 2014-07-31

**Authors:** Meghnath Dhimal, Ishan Gautam, Aljoscha Kreß, Ruth Müller, Ulrich Kuch

**Affiliations:** 1 Nepal Health Research Council (NHRC), Ministry of Health and Population Complex, Kathmandu, Nepal; 2 Biodiversity and Climate Research Centre (BiK-F), Frankfurt am Main, Germany; 3 Institute for Atmospheric and Environmental Sciences (IAU), Goethe University, Frankfurt am Main, Germany; 4 Natural History Museum, Tribhuvan University, Swayambhu, Kathmandu, Nepal; 5 Institute of Occupational Medicine, Social Medicine and Environmental Medicine, Goethe University, Frankfurt am Main, Germany; Centers for Disease Control and Prevention, United States of America

## Abstract

**Background:**

Rapidly increasing temperatures in the mountain region of Nepal and recent reports of dengue fever and lymphatic filariasis cases from mountainous areas of central Nepal prompted us to study the spatio-temporal distribution of the vectors of these two diseases along an altitudinal transect in central Nepal.

**Methodology/Principal Findings:**

We conducted a longitudinal study in four distinct physiographical regions of central Nepal from September 2011 to February 2012. We used BG-Sentinel and CDC light traps to capture adult mosquitoes. We found the geographical distribution of the dengue virus vectors *Aedes aegypti* and *Aedes albopictus* along our study transect to extend up to 1,310 m altitude in the Middle Mountain region (Kathmandu). The distribution of the lymphatic filariasis vector *Culex quinquefasciatus* extended up to at least 2,100 m in the High Mountain region (Dhunche). Statistical analysis showed a significant effect of the physiographical region and month of collection on the abundance of *A. aegypti* and *C. quinquefasciatus* only. BG-Sentinel traps captured significantly higher numbers of *A. aegypti* than CDC light traps. The meteorological factors temperature, rainfall and relative humidity had significant effects on the mean number of *A. aegypti* per BG-Sentinel trap. Temperature and relative humidity were significant predictors of the number of *C. quinquefasciatus* per CDC light trap. Dengue fever and lymphatic filariasis cases had previously been reported from all vector positive areas except Dhunche which was free of known lymphatic filariasis cases.

**Conclusions/Significance:**

We conclude that dengue virus vectors have already established stable populations up to the Middle Mountains of Nepal, supporting previous studies, and report for the first time the distribution of lymphatic filariasis vectors up to the High Mountain region of this country. The findings of our study should contribute to a better planning and scaling-up of mosquito-borne disease control programmes in the mountainous areas of Nepal.

## Author Summary

Dengue fever, a viral disease transmitted by the bites of infected *Aedes aegypti* and *Aedes albopictus* mosquitoes, has been rapidly spreading in Nepal since it was first reported in this country in 2004. Similarly, lymphatic filariasis, a parasitic disease transmitted by *Culex quinquefasciatus* mosquitoes in Nepal, is a public health problem in terms of morbidity and impact on the social and economic status of poor people living in rural and slum areas. Evidence for more pronounced temperature rises in higher altitudes of Nepal and an increasing frequency of dengue fever and lymphatic filariasis cases reported from mountain areas, in the absence of recent data on the mosquito vectors of these diseases, prompted us to investigate their distribution and abundance in this country. In our study, we document the distribution of *A. aegypti* and *A. albopictus* from the lowlands up to 1,310 m altitude in Kathmandu, and the distribution of *C. quinquefasciatus* up to Dhunche (2,100 m altitude), the highest locality included in this study. The wide distribution of these important disease vectors in the mountains, previously considered non-endemic for dengue fever and lymphatic filariasis, calls for an extension and scaling-up of vector-borne disease surveillance and control programmes in Nepal.

## Introduction

Dengue fever (DF) is a mosquito-borne viral disease which has become a major international public health concern in recent years. Dengue virus (DENV), the causative agent of this disease, belongs to the genus *Flavivirus*, family Flaviviridae, and is transmitted by *Aedes* mosquitoes, especially by the yellow fever mosquito (*Aedes* [*Stegomyia*] *aegypti*) and the Asian tiger mosquito (*Aedes* [*Stegomyia*] *albopictus*) which are respectively considered to be its primary and secondary vectors in Southeast Asia [1], [2]. In the last five decades, the incidence of DF has increased 30-fold, and geographical expansions to new countries and, in the present decade, from urban to rural settings have occurred [3]. For example, in the World Health Organization (WHO) South-east Asia Region (SEARO), the area with autochthonous DENV transmission has extended to the sub-Himalayan foothills of Bhutan and Nepal since 2004 and 2006, respectively [3], [4]. In the past, DF had often been considered a public health problem of lesser concern because of its low mortality rate and an infrequent occurrence of epidemics [5]. However, rapid economic development and urban growth in developing countries with a lack of careful planning of housing, water resources, sewage and waste management, along with the globalization of trade and travel, have since contributed to rendering DF the most important mosquito-borne viral disease of humans [5]. Despite progress with the development and clinical trials of vaccines against DENV infection, no such vaccine is available on the market yet [6], and there is no specific antiviral treatment either. Thus, controlling the population of dengue vector mosquitoes, especially *A. aegypti* and *A. albopictus*, and limiting their dispersal to new regions remains crucial for the prevention and control of DENV transmission [4].

The first case of DF in Nepal was a Japanese volunteer in 2004 [7], and the presence of the disease in the country was officially confirmed after an outbreak in 2006 [8]. In this first DF outbreak, 32 confirmed cases were reported, followed by 27 cases in 2007, 10 in 2008, 30 in 2009, and 917 cases including five deaths in 2010 when a major epidemic occurred in the Chitwan and Rupandehi districts of central and western Nepal, respectively [9]. More importantly, laboratory tests confirmed the presence of all four DENV serotypes in Nepal which portends the emergence of more severe DENV infections like dengue haemorrhagic fever (DHF) and dengue shock syndrome (DSS) [8]. During the 2010 epidemic, a DF case with no recent travel history to known DF affected areas was for the first time found in Kathmandu [10]. This case along with reports of *A. aegypti* collections in Kathmandu [11] suggest that local transmission of DENV has already occurred in Kathmandu, the capital city of Nepal which is located at altitudes around 1,300 m above sea level (asl). Furthermore, DF cases admitted to Sukraraj Tropical Diseases Hospital in 2010, one of the referral hospitals of Kathmandu, came from 24 of the 75 districts of Nepal, spanning the geographical regions of the Terai, Siwalik and Middle Mountains and indicating a rapid spread of DENV in the country [10]. Entomological investigations from the 1950s on had demonstrated the presence of *A. albopictus* in the Terai plains, Siwalik hills and Middle Mountain region [12], [13], but *A. aegypti* had not been found at the time. Entomological surveys carried out in 2006 and following years showed that *A. aegypti* was now present locally [8], [11]. However, apart from a few recent surveys of mosquito larvae in Kathmandu valley [11], [14], no detailed entomological studies on DENV vectors in Nepal have been conducted. Moreover, it remained to be determined how frequently the immature stages that had been found actually emerge as adult mosquitoes in the mountains compared to the Terai lowlands of Nepal.

Lymphatic filariasis (LF), one of the oldest known and most devastating neglected tropical diseases, is caused by three species of parasitic worms (*Wuchereria bancrofti*, *Brugia malayi* and *Brugia timori*). Globally about 90% of all LF infections are caused by *W. bancrofti* [15]. These parasites are transmitted between humans by different mosquito species depending on the geographical setting, e.g., species of the genus *Culex* mainly in urban and semi-urban areas of Asia, *Anopheles* species mainly in rural areas of Africa and *Aedes* species mainly in disease endemic islands of the Pacific, and by various species of the genus *Mansonia* [15]. In 2011, approximately 120 million people in 73 countries were infected with LF, and more than 1.3 billion were at risk [16]. The WHO SEARO shared the highest burden of LF among the six WHO regions, accommodating 65% of the global population at risk and 50% of the infected cases (60 million) [16].

In Nepal, LF is a major public health problem in terms of morbidity, primarily due to lymphedema, which causes the swelling of arms, legs, breasts and genitalia, and hydrocele, the swelling of the scrotum in male patients, hindering the socio-economic development in disease endemic areas and leading to social exclusion and stigmatization [17]. The disease is poverty-related, and marginalised groups and the poorer sector of the communities are predominantly affected [17], [18]. An LF mapping of Nepal using immunochromatographic card tests (ICT) was completed in 2005. It revealed that the disease is endemic in 60 out of the 75 administrative districts ranging from the lowlands (around 90 m) to more than 1,765 m above sea level in the mountains [17]. *Wuchereria bancrofti*, the only recorded LF parasite in Nepal, has been detected in all of these 60 endemic districts and is reported to be transmitted by *Culex quinquefasciatus* mosquitoes in Nepal [17]. The Epidemiology and Diseases Control Division (EDCD) under the Department of Health Services (DoHS) of the Ministry of Health and Population of the Government of Nepal has formulated a National Plan of Action (2003–2020) with an aim to eliminate LF in Nepal by 2020 [17]. Accordingly, the national LF programme has started the interruption of transmission by yearly mass drug administration (MDA) using a two drug regimen (diethylcarbamizine [6 mg/kg] plus albendazole [400 mg] in a single dose) in Parsa district in 2003. Since then the programme has expanded gradually to other endemic districts. In five districts, the MDA was completed and stopped after five rounds while the programme is going on in another 46 endemic districts with plans to start MDA in the remaining nine endemic districts in 2014 and achieve <1% microfilaria prevalence by 2018 [17]. However, a low acceptance of MDA due to severe adverse effects in some people, low coverage in urban areas and the movement of people between endemic and non-endemic areas pose challenges for LF elimination in Nepal [17]. Although MDA continues to be the mainstay for interrupting LF transmission, vector control is increasingly recognized as an important supplementary strategy for achieving LF elimination goals [19], [20]. An earlier mapping of LF in 37 districts of Nepal in 2001 had shown a high prevalence in Middle Mountain districts including Kathmandu [21]. The locality with the highest altitude sampled in that study was located at 1,400 m. Similarly, a sentinel surveillance study conducted in 2007 revealed that the highest microfilaria infection rates occurred in High Mountain district [22]. Therefore, there is a need of further study to better define the geographical limits of the endemic zone of LF in high mountain areas. However, to the best of our knowledge, no such work has yet been reported from Nepal. Mosquito vector monitoring and control play a complementary role in LF elimination at two stages: by adding to the reduction of microfilaria density and prevalence due to an active reduction of transmission during MDA, and by preventing recurrence or new infections in the surveillance phase after transmission has been interrupted [20]. Furthermore, monitoring the mosquito vectors and their infection status helps to identify new endemic areas and to make informed decisions about the need for an extension and scaling-up of MDA and vector control programmes in Nepal.

There is increasing evidence that global change, including climate change, affects the geographical distribution of vector-borne diseases. For example, model projections show a potential increase in the latitudinal and altitudinal range of DF as well as an increase in the potential duration of the transmission season and epidemic potential in temperate regions [23], [24]. The analysis of maximum temperature data from 49 stations in Nepal for the period 1971–1994 reveals warming trends after 1971, ranging from 0.06°C year^−1^ in most of the Middle Mountain and High Mountain regions, while the lowland regions of the Siwalik hills and Terai plains show warming trends of less than 0.03°C year^−1^ or even cooling trends (−0.03°C year^−1^) [25]. An extended analysis of temperature data after 1994 also shows a continuing warming trend without decrease [26]. A recent analysis carried out with data of 13 mountain stations of Nepal (1980–2009) shows that the maximum temperature and annual temperature are likely to increase while variability is too high to estimate a trend of the minimum temperature [27]. However, a general warming trend of minimum temperatures is observed in Nepal when data of the 36 years between 1971–2006 are analysed, showing a higher rate in mountain regions and lower rates in lowland Terai regions [28]. These trends of increasing temperature at higher altitudes of Nepal and the frequent reports of DF and LF cases from the mountain region of this country prompted us to conduct a study on the occurrence and abundance of DENV and LF vectors along an altitudinal transect of Nepal. This article documents the spatial and temporal distribution of the important vectors *A. aegypti*, *A. albopictus* and *C. quinquefasciatus* in central Nepal.

## Methods

### Study area

Nepal is a landlocked country that is administratively divided into five development regions (equivalent to provinces), 14 zones and 75 districts. The central development region (referred to later as central Nepal) consists of 19 districts and covers an area of 27,410 km^2^. As per the latest census of 2011, the total population of this region is 9,656,985; this constitutes the highest population density of the country with 352 people per km^2^ (the national population density is 180 per km^2^) [29]. Most urban areas (20 of total 58) are located in central Nepal. Central Nepal comprises all types of physiographical regions: the lowlands of the Terai and Siwalik, Middle Mountain, High Mountain and High Himalayan regions [30].

**Figure 1:**
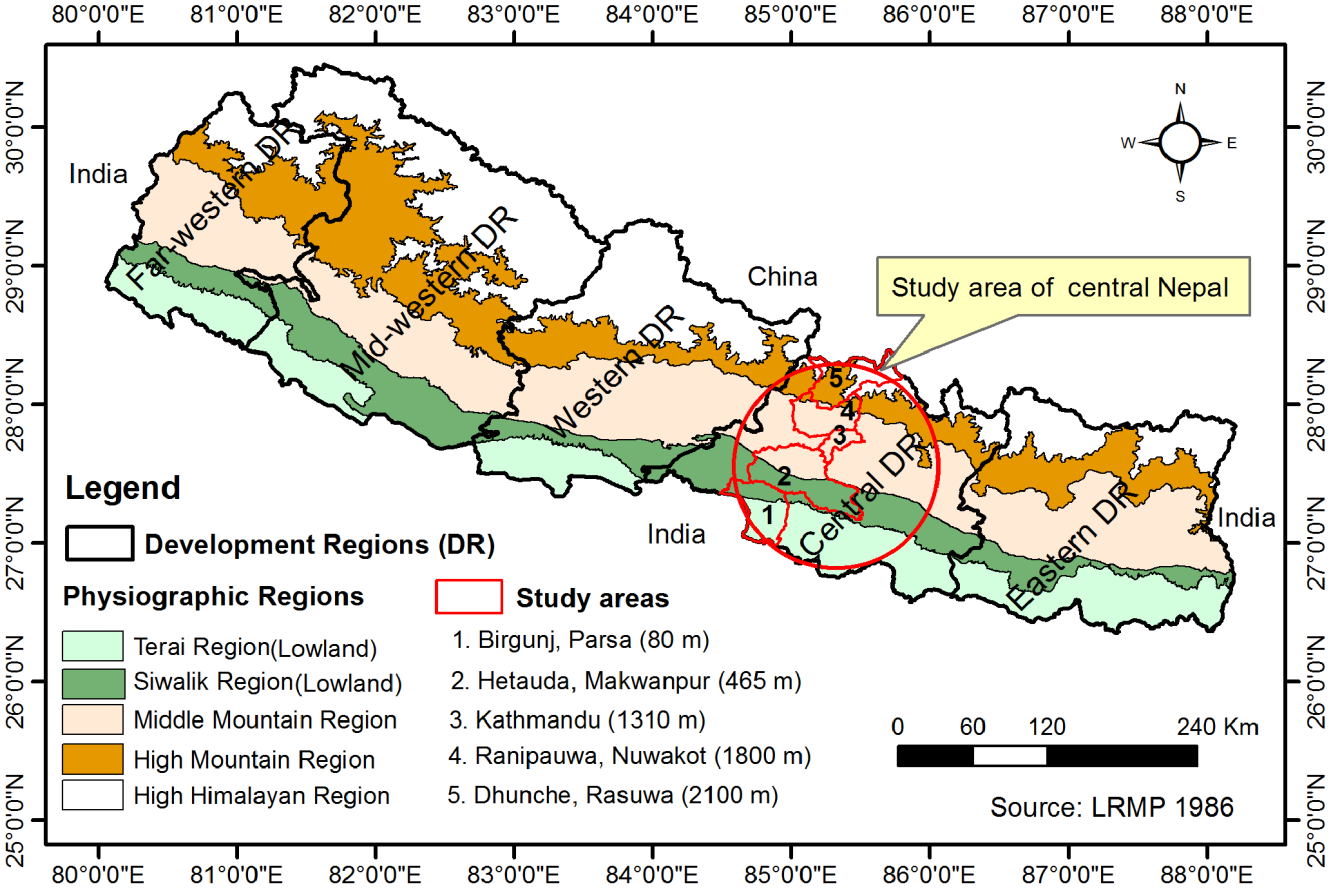
Map of the study areas. The map shows physiographic regions, development regions and study sites along an altitudinal transect from the lowlands (Birgunj; 80 m above sea level) to the High Mountain region (Dhunche; 2,100 m above sea level) in central Nepal.

For the entomological survey, the urban sites Birgunj (26°59′59″N, 84°52′00″E, 80 m asl), Hetauda (27°25′02″N, 85°01′59″E, 465 m asl) and Kathmandu (27°41′59″N, 85°20′01″E, 1,310 m asl) were selected from the Terai, Siwalik and Middle Mountain regions, respectively. In addition, the densely populated rural sites Ranipauwa (27°49′54″N, 85°14′21″E, 1,825 m asl) and Dhunche (28°06′45″N, 85°17′45.50″E, 2,100 m asl) were selected from the High Mountain region. The altitudes of the study sites range from less than 85 m asl at the southern border with India to more than 2,100 m asl near the border with China in the north, representing a vertical cross-section of each physiographical region of the country to the exception of the High Himalayan region which we did not attempt to sample for the present study. The sites are connected to each other by highways and hence population mobility along the transect is high. The geographical locations of the study sites are presented in Figure 1; their socio-demographic characteristics are provided in Dhimal et al. [31].

Birganj is a sub-metropolitan city, headquarters of Parsa district and border town in southern Nepal. According to the 2011 census of Nepal, Birganj has a population of 139,068. It is located 91 km (airline distance) south of the capital Kathmandu and 3 km north of the border with the Indian state of Bihar. Being the main entry point to Nepal from the Indian cities Patna and Kolkata, it is also known as the gateway to Nepal. As a large part of the country's imported goods enters Nepal through Birganj, the town has a significant economic importance. The climate of Birganj is considered to be sub-tropical.

Hetauda is a municipal area and headquarters of Makwanpur district within the Siwalik region. It has 85,653 inhabitants and is one of the important industrial areas of Nepal. The airline distance between Hetauda and Kathmandu is 43 km. Hetauda is situated in a unique geographical structure called ‘doon’, which means valley-like geography, and is surrounded by the Middle Mountains in the north and the low rolling Siwalik range with sub-tropical climate in the south.

Kathmandu is the capital of Nepal and, with close to one million inhabitants, its largest metropolitan city. The city lies in a bowl-shaped valley in central Nepal that is surrounded by four mountains: Shivapuri, Phulchowki, Nagarjun and Chandragiri. Among the five major climatic zones of Nepal, Kathmandu valley belongs to the warm temperate zone.

Ranipauwa is a settlement with 7,320 inhabitants in the Kakani Village Development Committee of Nuwakot district in central Nepal. The airline distance between Ranipauwa and Kathmandu is 20 km. It has a cool temperate climate, is a popular picnic and trekking destination, and has a good road connection.

Dhunche, the headquarters of Rasuwa district, is located in the High Mountain region, has a cool temperate climate and is home to about 2,744 people. The airline distance of Dhunche from Kathmandu is 45 km. In the north, Rasuwa district is bordered by the Langtang mountain range and national park. This area has a good road connection from Kathmandu to China border.

### Entomological survey

The longitudinal entomological survey covered five administrative districts of central Nepal representing four physiographical regions (the Terai and Siwalik lowlands, Middle and High Mountain regions). In each region, adult mosquitoes were collected for half a year from September 2011 to February 2012 (end of monsoon, post-monsoon, and winter seasons) using BG-Sentinel traps (Biogents, Regensburg, Germany) and Centers for Disease Control (CDC) light traps (BioQuip Products, USA). Mosquito eggs were collected using ovitraps.

Adult mosquitoes were captured in each site using twelve BG-Sentinel mosquito traps (two traps per site per month). As one BG-Sentinel trap in Hetauda failed in February 2012, a total of 59 BG-Sentinel trap collections were performed. Each trap was operated for 24 hours using a 12 V battery at each time and site with BG-Lure attractant (Biogents, Regensburg, Germany) according to the manufacturer's protocol. In addition, we used dark activated CDC light traps fitted with double ring fine mesh collection bags (BioQuip Products, USA) and operated for 12 hours for the outdoor collection of mosquitoes. Two BG-Sentinel traps and one CDC light trap were randomly allocated to three fixed places in each of the study sites and operated monthly in the same places. We assumed the maximum adult longevity of *A. aegypti*, *A. albopictus* and *C. quinquefasciatus* to be three weeks [32] and that adult mosquitoes captured on consecutive sampling dates should have emerged between capture dates. Thus, this sampling strategy was expected to avoid generation overlap and temporal auto-correlation.

The ovitraps used in this survey consisted of 300 ml cups made of black plastic. The upper three quarters of the inside of the cup were lined with one layer of an inexpensive, locally purchased, indigo-coloured low thread count cotton fabric which was held in place by a single paper clip [33]. The fabric was labelled with the house number and location of the ovitrap (indoors or outdoors) using a permanent marker. In addition, the same plastic cups were used to make ‘traditional’ ovitraps with a single, plain wooden tongue depressor paddle instead of fabric as the oviposition substrate. The wooden paddle was labelled on its back with the house number and location of the ovitrap using a permanent marker. Both types of ovitrap were filled to three quarters with distilled water and deposited outside of 30 houses in low-lying areas of the study sites that were protected from sun and rainfall by a roof or other cover. We then recorded geographical coordinates of each sampling point using portable global positioning system (GPS) devices (Garmin eTrex H). Permission was obtained from the inhabitants of the houses before ovitraps were placed. Ovitraps were monitored every month from September 2011 to February 2012 on the dates when BG-Sentinel and CDC light trap collecting was performed. Mosquito eggs were counted under a stereo microscope, identified to genus level, and the number of eggs per ovitrap and day was calculated. As the proportion of other *Aedes* spp. among our adult mosquito collections was very low in the study sites, it was assumed that most of the collected eggs that looked like *A. aegypti* and *A. albopictus* eggs had indeed been deposited by these species. However, no efforts were made to verify this identification or distinguish between the eggs of *A. aegypti* and *A. albopictus*, e.g., by molecular analysis or hatching and raising them to adulthood.

### Meteorological data

Daily records of the average minimum and maximum temperature, morning and evening relative humidity and rainfall from August 2011 to February 2012 for each study site were obtained from the Department of Hydrology and Meteorology, Ministry of Science, Technology and Environment, Government of Nepal. The meteorological stations were within 1–5 km from mosquito collection spots. Using these data, we computed the derived variables adjusted rainfall (ADJRAIN) and adjusted temperature (ADJTEMP) [32]. ADJTEMP is the mean of the daily average temperatures during the three weeks prior to the collection date, and ADJRAIN is the accumulated rainfall during the third and second week before each mosquito collection date. Furthermore, we also calculated the adjusted relative humidity (ADJRH) using the same procedure as for the ADJTEMP.

### Dengue fever and lymphatic filariasis data

In the absence of an active case detection surveillance system of DENV infection in Nepal, we collected reported DF cases from the passive surveillance system and published literature. Dengue fever cases were reported from all three study areas (Terai, Siwalik and Middle Mountain regions) of central Nepal except the High Mountain region. Lymphatic filariasis cases were recorded only during campaign programmes for MDA which take place in selected districts each year and during the mapping phase of the LF elimination program, and were reported from all study sites except Dhunche, Rasuwa district.

### Ethics statement

Ethical approval for conducting this study was granted by the Ethical Review Board (ERB) of the Nepal Health Research Council. Stakeholders at the central level as well as local authorities were briefed about the objectives and procedures of the study before the beginning of the surveys. Oral informed consent to install traps and collect mosquitoes was obtained from all owners of households and their premises. Taking oral consent only was approved by the ERB. The identity of patients was not disclosed in the process of data transfer or analysis.

### Data processing and analysis

The data were entered into Microsoft Excel spreadsheets and further cleaned and manually edited if necessary. The data were then transferred to and analysed using R computing software [34]. The proportion of female mosquitoes of each species in each trap was calculated using an exact binomial test with female and male as outcomes. The GPS coordinates of trap placement locations and of those traps that were found positive were projected onto maps with ArcGis software (ArcGis10, ESRI). In most cases, the data for comparing the mean abundance of each species among study groups were not normally distributed and primarily right skewed. Therefore, generalized linear models (GLM) were fitted assuming negative binomial distribution and a log link function for each species using the “MASS” package in R [35]. The negative binomial distribution model is reported to be a robust analysis especially with respect to count data sets [36]–[38]. The full model was fitted including all possible interactions of selected variables. The selected predictor variables were trap type, month of collection and physiographic region. We fitted separate models for each species, mean abundance, and eggs per ovitrap as response variables and the trap type, month of collection and physiographic region as predictor variables.

We also calculated Spearman's rank correlation between the abundance of each mosquito species in each trap and meteorological variables. The resulting correlation coefficients showed that meteorological variables were better correlated with the number of *A. aegypti* in BG-Sentinel traps and the number of *C. quinquefasciatus* in CDC light traps but not significantly correlated with *A. albopictus* numbers in either trap. Therefore we further fitted generalized linear models for predicting the number of mosquitoes in each trap using the derived meteorological variables. We used Akaike's information criterion (AIC) to select the final model using an automated step function. When comparing two models, the smaller AIC was accepted as indicating the better fit [39]. We assessed multicollinearity of predictors for each model using variance inflation factors (VIFs). The VIFs for all predictor variables were less than 2.0. We compared the fit of models with (full) and without (reduced) the term of interest using an F-ratio test statistics [40]. Pearson's as well as deviance residuals were calculated for model checking and standardized residuals were plotted to find evidence whether the model assumptions were met or not. If the final model included a variable with more than two levels, Tukey's multiple comparisons were applied using the “multcomp” package in R [41]. Differences between groups were considered to be significantly different at a family error rate of p<0.05. The parameter coefficients and their 95% confidence intervals (CI) were transformed into original scale using exponential functions for easy interpretation of results. Effects display graphs were generated using “effects” package in R for GLM [42] and GraphPad Prism for few figures.

## Results

### Species and sex composition and abundance of mosquitoes collected in BG-Sentinel and CDC light traps in central Nepal

From September 2011 to February 2012, a total of 1,164 mosquitoes from the genera *Aedes*, *Anopheles*, *Armigeres* and *Culex* were captured in 59 BG-Sentinel trapping sessions (eight species) and 747 mosquitoes in 30 CDC light trap sessions (seven species) (Table 1). The GPS points of the collection sites and number of mosquitoes per month per site by trap type is provided in Table S1. From the viewpoint of LF and DENV transmission, *C. quinquefasciatus*, *A. albopictus* and *A. aegypti* are the most important vector species in this geographic setting. *Culex quinquefasciatus* was the predominant species in both trap types. Apart from *Culex vishnui*, all of the seven species captured in CDC light traps were also commonly caught in BG-Sentinel traps.

**Table 1:** Species and sex composition and abundance of mosquitoes collected in BG-sentinel and CDC light trap in central Nepal.

Species	Trap method									
	BG-sentinel trap (n = 59)					CDC light trap (n = 30)				
	Total (%)	Male	Female	95% CI for proportion of female	Sex ratio p-value	Total (%)	Male	Female	95% CI for proportion of female	Sex ratio p-value
*Aedes aegypti*	344 (29.6)	110	234	0.68 (0.62–0.73)	<0.001	7 (0.9)	2	5	0.71 (0.29–0.96)	0.45
*A. albopictus*	67 (5.8)	15	52	0.77 (0.66–0.87)	<0.001	10 (1.3)	3	7	0.7 (0.34–0.93)	0.34
Other *Aedes* spp.	13 (1.1)	2	11	0.84 (0.54–0.98)	<0.05	1 (0.1)	1	0	0 (0–0.97)	1
*Anopheles culicifacies*	1 (0.1)	0	1	1 (0.02–1)	1	3 (0.4)	0	3	1 (0.29–1)	0.25
*Armigeres* spp.	17 (1.5)	7	10	0.58 (0.33–0.81)	0.62	5(0.7)	1	4	0.8 (0.28–0.99)	0.37
*Culex fuscocephala*	5 (0.4)	0	5	1 (0.48–1)	1	5 (0.7)	3	2	0.4 (0.05–0.85)	1
*C. quinquefasciatus*	715 (61.4)	181	534	0.75 (0.71–0.78)	<0.001	716 (95.9)	158	558	0.78 (0.75–0.81)	<0.001
*C. vishnui*	2 (0.2)	0	2	1 (0.15–1)	0.5	0 (0)	0	0	ND	ND
Number of all mosquitoes	1164 (100)	315	849			747 (100)	168	579		

ND means not determined.

Overall, more than 68% of all mosquitoes caught with BG-Sentinel traps (except *Armigeres* spp.) were female (Table 1). In the case of *Anopheles culicifacies*, *C. vishnui* and *Culex fuscocephala*, only females were captured with BG-Sentinel traps during the study period. Similarly, significantly higher female catches (>70%) were recorded in CDC light traps except for *C. fuscocephala* (Table 1). Only one male of *Aedes* sp. and only three females of *Anopheles culicifacies* were recorded in CDC light traps.

**Figure 2:**
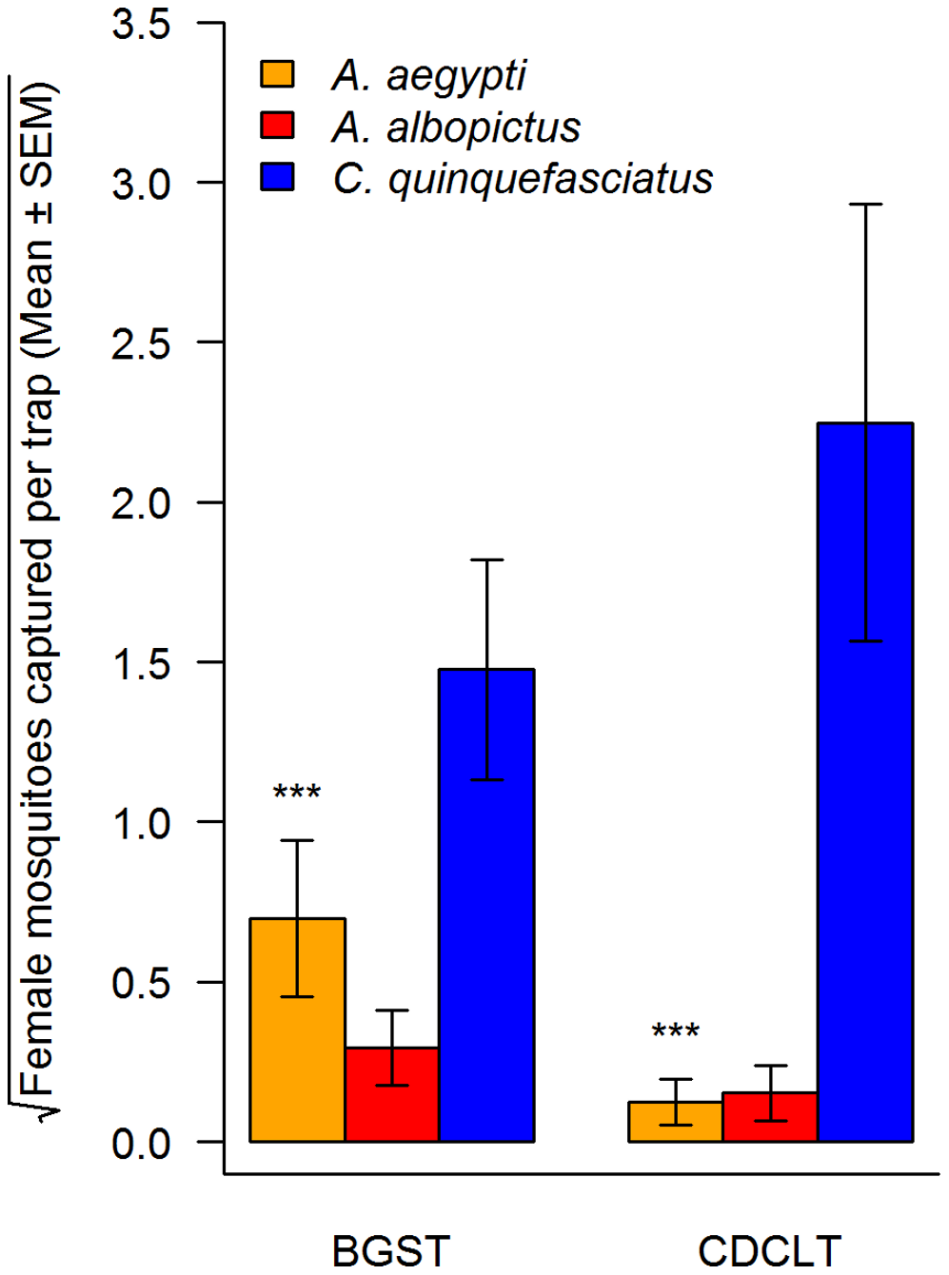
Number of female mosquitoes [mean ± SEM] collected in central Nepal with different trap types. The number of females per trap was square root transformed to visualize the data more clearly. BGST - BG-Sentinel trap, CDCLT - CDC light trap, n _(BG-Sentinel trap)_ = 59, n_(CDC light trap)_ = 30. The symbol *** indicates significant different from each other at a family error rate of p<0.001.

*Aedes aegypti*, *A. albopictus* and *C. quinquefasciatus* were the predominant species in both trap types with significantly higher proportions of females of all three species in BG-Sentinel traps and only *C. quinquefasciatus* females in CDC light traps (Table 1). Therefore, we included only females of these species in subsequent analyses. The mean abundance of females of these three species by trap type is shown in Figure 2.

### Effects of the trapping method, month and region

**Figure 3:**
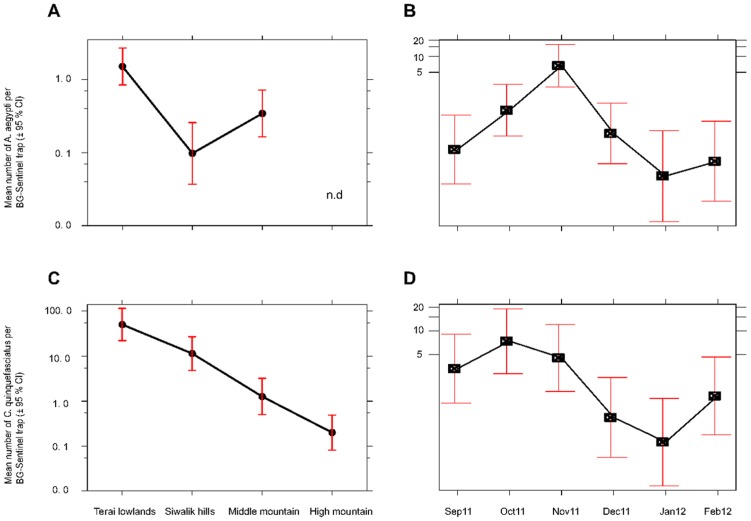
Effect plots for *Aedes aegypti* and *Culex quinquefasciatus* mean abundance in central Nepal. Panels A and B show the effect of physiographic regions and months of collection, respectively, on the mean abundance of female *A. aegypti*; panels C and D show the effect of physiographic region and month of collection on the mean abundance of female *C. quinquefasciatus* (pooled data from both trap types). Mosquito abundance is displayed in log scale. *Aedes aegypti* was not recorded in the High Mountain region and not determined (n.d).

Throughout the study period, *A. aegypti* and *C. quinquefasciatus* were continuously recorded in central Nepal whereas *A. albopictus* was not trapped in December. Trap method, month and region were not significant variables for predicting the mean number of *A. albopictus*. The effects of trap method (F = 20.91; df = 1; p<0.001), region (F = 10.89; df = 2; p<0.001) and month (F = 12.82; df = 5; p<0.001) were significant variables for predicting the mean number of female *A. aegypti*. Compared to CDC light traps, BG-Sentinel traps collected 19 times more female *A. aegypti* (95% CI = 3.7–101.3; p<0.001) (Table S2). Adult *A. aegypti* and *A. albopictus* were collected from the lowland Terai, Siwalik and Middle Mountain regions up to 1,310 m asl. The highest abundance of female *A. aegypti* was recorded in lowland Birganj followed by the Middle Mountains in Kathmandu (Figure 3A). The abundance of female *A. aegypti* significantly decreased in the Siwalik (β = 0.07; 95%CI = 0.01–0.31; p = 0.001) and Middle Mountain regions (β = 0.23; 95%CI = 0.06–0.82; p = 0.024) compared to the lowlands of the Terai. Similarly, compared to September, the abundance of female *A. aegypti* increased significantly reaching its peak in November (β = 36.98; 95%CI = 5.56–245.99; p<0.001) and then declined abruptly (Figure 3B). The regression model for predicting female *A. aegypti* mean abundance using categorical explanatory variables is provided in Table S2.

For predicting the mean number of female *C. quinquefasciatus*, only region (F = 21.34; df = 3, p<0.001) and month (F = 12.82; df = 5, p<0.001) were significant variables. Compared to the Terai lowlands, the abundance of female *C. quinquefasciatus* significantly decreased in the Siwalik (β = 0.27; 95%CI = 0.06–0.84; p = 0.026), Middle Mountain (β = 0.23; 95%CI = 0.06–0.82; p<0.001) and High Mountain regions (β = 0.004; 95%CI = 0.001–0.015; p<0.001) (Figure 3C). The mean abundance of female *C. quinquefasciatus* increased reaching its peak in October and then gradually declined (Figure 3D). The regression model for predicting female *C. quinquefasciatus* mean abundance is given in Table S3. No significant interactions were identified between predictor variables in either regression model. In addition, there was a significant effect of only region (F = 3.31; df = 3; p<0.01) on the mean number of *Aedes* eggs per ovitrap.

The highest mean number of female *A. albopictus* was recorded in Birganj (2.58±1.74 individuals [±SE]) followed by Kathmandu (1.5±0.94 individuals). The chi-square test indicated a significant association between abundance of female *A. albopictus* and region (χ^2^ = 13.45; df = 2; p = 0.001), month (χ^2^ = 91.40; df = 5; p<0.001) and trap method (χ^2^ = 34.32; p<0.001). In the High Mountain locations Ranipauwa and Dhunche, however, *A. aegypti* and *A. albopictus* were not recorded.

**Figure 4:**
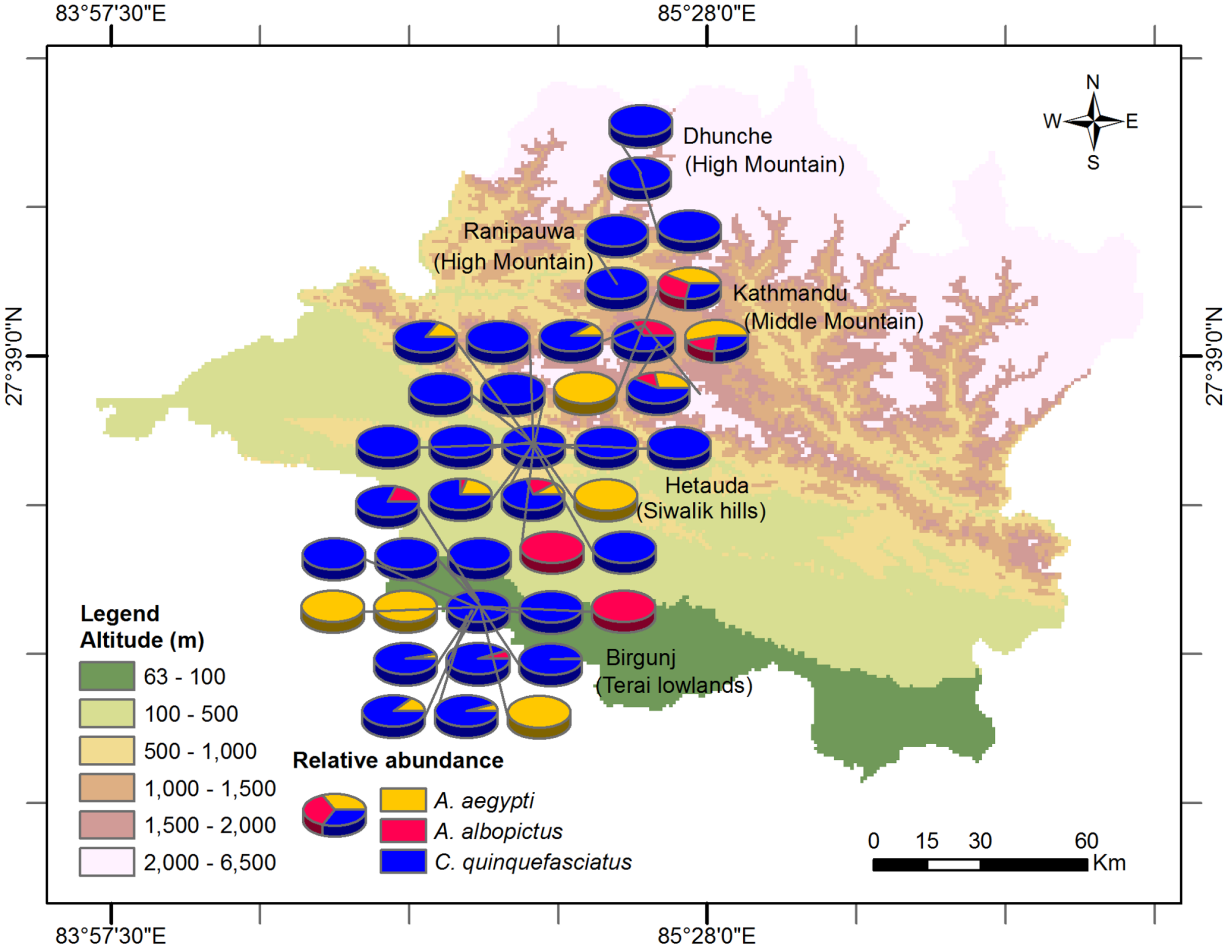
Relative abundance of collected female *Aedes aegypti*, *Aedes albopictus* and *Culex quinquefasciatus* in central Nepal. Each pie-chart represents positive traps for mosquitoes captured either by BG-Sentinel traps or CDC light traps.

Among the *Aedes* mosquitos, *A. aegypti* was the most abundant species captured in all of the regions except the High Mountain locations where both *A. aegypti* and *A. albopictus* were not found during our survey. Interestingly, the frequency of co-occurrence of *A. aegypti* and *A. albopictus* in traps was very low indicating that one species may dominate over the other locally (Figure 4).

### Effect of rainfall, temperature and relative humidity on the abundance of adult mosquitoes and number of eggs

**Figure 5:**
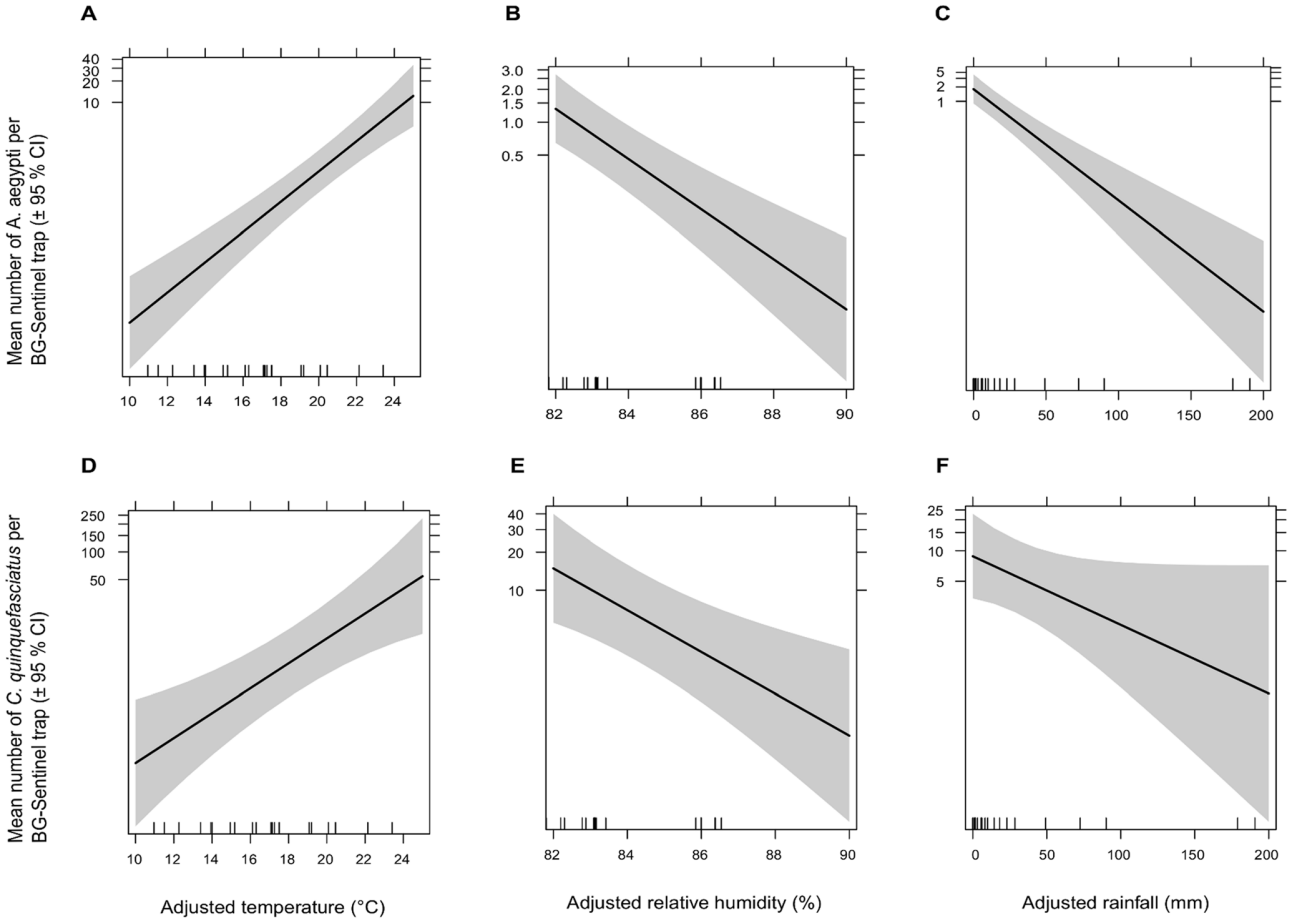
Effect plots of meteorological factors for *Aedes aegypti* and *Culex quinquefasciatus* mean abundance in central Nepal. Panels A, B and C show the effects of adjusted mean temperature (°C), adjusted relative humidity (%) and adjusted rainfall (mm), respectively, on the number of female *Aedes aegypti* per BG-Sentinel trap. Panels D, E and F show the effects of adjusted mean temperature (°C), adjusted relative humidity (%) and adjusted rainfall (mm), respectively, on the number of female *Culex quinquefasciatus* per CDC light trap. Mosquito numbers are displayed in log scale. The effect of adjusted rainfall on *C. quinquefasciatus* abundance was not significant but remained in the final model.

The generalized linear model with ADJRAIN (mm), ADJTEMP (°C) and ADJRH (%) as covariates for the mean abundance of female *A. aegypti* per BG-Sentinel trap indicated significant effects of these three variables (Figure 5). Each degree rise in ADJTEMP increased female *A. aegypti* abundance (β = 1.63; 95%CI = 1.34–1.98; p<0.001); increased ADJRAIN reduced abundance (β = 0.94; 95%CI = 0.92–0.97; p<0.001) and increased ADJRH also reduced abundance (β = 0.59; 95%CI = 0.44–0.77; p<0.001). Likewise, an increase of the ADJRAIN had a negative effect (β = 0.98; 95%CI = 0.96–1.00; p = 0.050), ADJTEMP had a significantly positive effect (β = 1.36; 95%CI = 1.16–1.60; p<0.001), and ADJRH had a significantly negative effect (β = 0.68; 95%CI = 0.54–0.85; p<0.001) on the number of female *C. quinquefasciatus* per CDC light trap. Moreover, all these three covariates had significant effects on the number of *C. quinquefasciatus* female per BG-Sentinel trap (data not shown). The ADJRH had significantly negative effects on the mean number of *Aedes* eggs per ovitrap (β = 0.83; 95%CI = 0.71–0.97; p<0.001). We did not find any significant effect of rainfall and temperature on the number of *Aedes* eggs per ovitrap (p>0.05). The number of *Aedes* eggs in ovitraps was positively correlated with the number of female *A. aegypti* per BG-Sentinel trap (r_s_ = 0.64; p<0.001). The number of *Aedes* eggs per ovitrap was likewise positively (but not significantly) correlated with the number of *A. albopictus* per BG-Sentinel trap (r_s_ = 0.20; p>0.05).

## Discussion

The present entomological study of DF and LF vectors in four distinct physiographical regions of central Nepal from September 2011 to February 2012 documented differences in their spatial and temporal dynamics. The DENV vectors *A. aegypti* and *A. albopictus* were found from the lowlands up to the Middle Mountain region (1,310 m asl) in central Nepal. *Culex quinquefasciatus*, the principal vector of *W. bancrofti* filariasis in Nepal [12], [43], was found throughout the studied longitudinal gradient from 80 to 2,100 m asl and was constantly abundant in the six-month trapping period. The presence of these vectors and reports of cases of DF up to the Middle Mountain region and LF up to the High Mountain region suggest that DENV and the LF parasite *W. bancrofti* are potentially established in central Nepal.

### Trap efficiencies

This is the first longitudinal entomological survey of adult *Aedes* and *Culex* mosquito vectors of DENV and LF using BG-Sentinel together with CDC light traps in Nepal. As the use of CO_2_ for monitoring mosquitoes is difficult in Nepal because of logistic and economic reasons, one aim of our field study was to test the regular monitoring of DF and LF vectors with BG-Sentinel and CDC light traps without the use of CO_2_ as an attractant. We captured a substantial number of various mosquito species in both traps, and the species captured were common in both traps with only one additional species (*C. vishnui*) in BG-Sentinel traps. *Culex quinquefasciatus* was the predominant species in both types of traps (Table 1), but the mean number of female *C. quinquefasciatus* per trap was nearly two times higher in CDC light traps than in BG-Sentinel traps (Figure 2). These findings suggest that CDC light traps without CO_2_ and BG-Sentinel traps with BG-Lure attractant can both be used in Nepal for monitoring adult *C. quinquefasciatus*.

On the other hand, BG-Sentinel traps with BG-Lure attractant were much more efficient capturing *A. aegypti* and *A. albopictus* than CDC light traps. The trapping method obviously had a significant effect on the mean number of female *A. aegypti* caught, and the abundance of female *A. aegypti* was 19 times higher in BG-Sentinel than in CDC light traps. This indicates that, although both traps were able to capture *A. aegypti*, the BG-Sentinel trap was the more efficient trap for collecting *A. aegypti* in our study areas. The black and white visual target and contrast characterizing the BG-Sentinel trap are factors that contribute to the successful collection of this diurnal mosquito species. Also, the BG-Sentinel trap does not need CO_2_ for collecting DENV vectors and its BG-Lure attractant remains active for several months. Thus, this trap can be used for the long-term monitoring of DENV vectors and certain other mosquitoes of medical importance that it reliably attracts [44]. As previously demonstrated in other studies [44]–[47], the BG-Sentinel trap equipped with BG-Lure attractant was the superior surveillance tool for *A. aegypti* populations in our study compared to CDC light traps. In agreement with findings from Puerto Rico [32] and Brazil [46], a higher number of *C. quinquefasciatus* than *A. aegypti* were trapped in BG-Sentinel traps in our study. Although the difference was statistically not significant due to the overall relatively low number of *A. albopictus* that were collected along our transect, the BG-Sentinel traps also collected more *A. albopictus* compared to the CDC light traps in the present study which is consistent with previous reports [48]–[50].

The strongly significant positive correlation between the number of female *A. aegypti* caught in the BG-Sentinel trap and the number of *Aedes* eggs collected in the ovitraps, non-significant positive correlation between the number of female *A. albopictus* in BG-Sentinel traps and the number of *Aedes* eggs in ovitraps, and low number of other *Aedes* spp. collected in our study sites, suggest that the majority of *Aedes* eggs in the ovitraps might have been from *A. aegypti*. This positive correlation between the number of eggs and number of female *A. aegypti* in BG-Sentinel traps is similar to findings from Puerto Rico [32]. In addition, many *Culex* eggs which might have been mostly from *C. quinquefasciatus* were recovered from these ovitraps. This indicates that such simple ovitraps or similar devices may be useful tools for simultaneously monitoring populations of gravid female mosquitoes as indicators of potential DENV [32] and LF transmission in Nepal.

### Spatial distribution

Our study clearly demonstrated differences in the spatial distribution of these three important vector mosquito species and a strong effect of the physiographical region on the mean abundance of female *A. aegypti* and female *C. quinquefasciatus*. Similar findings on the effect of geographical region on the abundance of *A. aegypti* and *A. albopictus* have been reported from Vietnam [51] where *A. aegypti* predominated in urban-rural areas in the southern lowlands and *A. albopictus* in the northern region with more mountainous areas. In our survey, *A. aegypti* and *A. albopictus* were not collected in the High Mountain region which included some rural areas, and *A. aegypti* was predominant throughout the urban agglomerations of the Middle Mountain, Siwalik and Terai regions in Nepal (Figure 3). *Aedes albopictus* and *C. quinquefasciatus* had been recorded as early as the 1950s in Nepal [13] and were later recorded from the Terai lowlands, Siwalik hills (Makwanpur) and Middle Mountain regions (Kathmandu) [12]. In contrast, *A. aegypti* was recently introduced to Nepal where it was first recorded in a few cities in lowland areas near the southern border with India in 2006 [8]. In the Middle Mountain region of Nepal it was first found in Kathmandu in 2009 [11]. A study conducted in the Gharwal Himalayan region of neighbouring India reported the distribution of adult *A. albopictus* from 300–1,300 m, *A. aegypti* up to 800 m and *C. quinquefasciatus* up to 2,000 m asl [52], roughly similar to our distribution records. The high spatial heterogeneity of the three species in the present study is consistent with reports that DENV is highly focal in nature [53] and DF outbreaks closely linked to the abundance of *A. aegypti* [54]. In our study, a BG-Sentinel trap installed in the Terai captured 84 male and 151 female *A. aegypti* during 24 hours in November.

The fact that no adult *A. aegypti* and *A. albopictus* were collected in the High Mountain localities in our study suggests that both species are either not yet established at this altitude in this region, or could not be trapped due to their low population density during the study period. Other *Aedes* species were recorded up to the highest studied location at 2,100 m asl. The frequency of co-occurrence of both species in the Siwalik and Middle Mountain regions in the present study was consistent with findings from Vietnam [51]. Because of the low frequency of occurrence and low abundance of *A. albopictus*, no meaningful statistical analysis of this species could be performed. One possible explanation for the rarity of *A. albopictus* in this study might be our sampling in highly urbanized sites since *A. albopictus* has been reported to be mainly found in sub-urban and rural areas [55]–[57]. On the other hand, this species might be displaced by the newly introduced *A. aegypti* as has been reported in many studies in other South Asian countries [58]–[61]. In contrast, in settings with more temperate climate like the USA [62] where cold winters are a limiting factor for these mosquitoes, the recent establishment and spread of *A. albopictus* has largely displaced previously established *A. aegypti* populations, and *A. albopictus* has become the most abundant mosquito in man-made containers in much of the south-eastern USA [62]–[64].

### Temporal distribution

The effect of the month of collection was significant for the mean abundance of females of *A. aegypti* and the mean abundance of female *C. quinquefasciatus*. The mean abundance of *A. aegypti* and *A. albopictus* was higher in the end of monsoon and post-monsoon rainy seasons (September–November) than in the winter season (December–February) with a peak in November in each region except the High Mountains where these species were not recorded. The peak mean abundance of these two *Aedes* species in the month of November may, on the one hand, be attributed to conditions of only slight rainfall, a moderate mean temperature (20°C) and an optimal temperature range (10–30°C). In addition, by November large populations of these species could have built up since the preceding monsoon rainy season (June–August) using the numerous available water-filled containers. The populations of adult female *C. quinquefasciatus* peaked in October in the High Mountain region and in November in the Terai and Siwalik lowlands and Middle Mountains. In agreement with our findings, effects of the month or season of collection on the abundance of *Aedes* and *Culex* species elsewhere have been reported in many previous studies [51], [65]–[68].

### Effects of climatic factors on the abundance of mosquitoes and their eggs

Regional environmental conditions may strongly determine the local abundance and distribution of mosquito species. The meteorological factors adjusted temperature, rainfall and relative humidity had significant effects on the mean number of female *C. quinquefasciatus* collected per BG-Sentinel trap. However, rainfall was not significant predictor of the mean number of female *C. quinquefasciatus* per CDC light trap which might be because of small sample size (n = 30) compared to BG-sentinel trap (n = 59). Effects of temperature and rainfall on the population dynamics of *C. quinquefasciatus* observed along an altitude gradient in Hawaii [69] are consistent with our findings. *Culex quinquefasciatus* is a common domestic species whose preferred habitats range from clean freshwater to brackish, turbid and polluted water, and which is commonly found in ground pools, ditches, drains, sewerage, latrines, septic tanks and artificial containers such as discarded tires in Nepal [12]. Being highly anthropophilic, biting predominantly at night but also at daytime in dark rooms, and feeding both indoors and outdoors, it is an efficient vector for maintaining low levels of microfilaria within a population [19], [70]. Therefore, unplanned urbanization, poor sanitation and drainage systems and the expansion of transportation systems in rural areas and highlands including pronounced rise of temperature in mountain regions may have led to a present and future expansion of the distribution of LF vectors. Falling temperatures coupled to increasing elevation, on the other hand, have negative effects on mosquito survival and parasite development rates [71], [72]. Model projections show a wide distribution of LF in Africa, and that climate change and population growth will expand both the range and risk of LF infection in an endemic region of Africa [73]. Another study suggests that population growth rather than climate change is the dominant factor for predicting the prevalence and spread of LF on the African continent [74]. However, predicting the impact of climate change on LF is difficult owing to the chronic condition of this disease.

We found significant effects of adjusted temperature, relative humidity and rainfall on the mean number of female *A. aegypti* per BG-Sentinel traps which is consistent with previous findings [32]. These reports indicate that temperature, relative humidity and rainfall play a significant role for the abundance of these mosquito species but not necessarily as a direct driver. Important effects of temperature on these species and the diseases they can transmit are those that shorten the extrinsic incubation period of pathogens, lead to increases in biting frequency and extensions of the average life span of mosquitoes [75]–[77]. Hence, increasing temperature can make temperate regions of Nepal vulnerable to DF epidemic. Interestingly, we collected *A. aegypti* and *A. albopictus* in the BG-Sentinel traps in the Terai lowlands even when minimum temperatures had dropped to around 8°C suggesting a considerable adaptive capacity of local *A. aegypti* and *A. albopictus* populations to low temperatures. No *Aedes* species could be captured in the Middle Mountain collection sites after November when mean temperatures had dropped to around 10°C and minimum temperatures to around 2°C during a few days. However, a substantial number of *Aedes* eggs was recorded in ovitraps that had been set up indoors in these sites, indicating an ongoing reproductive activity of *A. aegypti* during the cold winter months. Thus, low temperatures outdoors, where the BG-Sentinel traps had been positioned, may have been compensated by an indoor environment with temperatures that were still sufficient for oviposition in another example of the ability of vector insects to exploit human activities to their advantage [78].

Rainfall can be an important abiotic factor for mosquitoes in areas where their breeding sites are produced by rainfall. However, the effect of rainfall is more complex. In some cases, increased rainfall may increase the vector population size by creating new or better larval habitats while excessive rain would eliminate habitats through flooding, thus decreasing the vector population [79], [80]. Drought events, while having a negative impact on natural breeding sites, can increase mosquito abundance by increasing man-made breeding sites created by household water storage [81]. In Nepal, the observed decline in the abundance of mosquitoes during the monsoon season which lasts from June to September (low abundance in September) might not only be attributed to the flushing of drains and the flooding of other outdoor breeding foci, but also to increased mortalities due to physical impact by heavy rain. During the post-monsoon season lasting from October to November with very low or no rainfall, we observed higher numbers of *A. aegypti*, *A. albopictus* and *C. quinquefasciatus* compared to September with high rainfall. In our study areas, many containers such as discarded tires, cemented tanks and metal drums with water provided suitable breeding sites in the post-monsoon season.

Entomological indices are used to predict dengue risk transmission in many studies. To prevent DENV transmission, Barrera et al. [32] suggested that the number of female *A. aegypti* per BG-Sentinel trap and the number of eggs per ovitrap should be maintained well below two and ten, respectively. Accordingly, Mogi et al. [82] did not report DHF cases in Chiang Mai, Thailand, when the number of eggs in ovitraps was less than two. In our study, we found more than two female *A. aegypti* per BG-Sentinel trap and more than ten eggs per ovitrap in the Terai and Middle Mountain regions indicating a risk of DENV transmission in both regions. Interestingly, DF cases have been reported from the Terai and Middle Mountain regions, but also from the Siwalik regions [83], [84] where the number of female *A. aegypti* per BG-Sentinel trap and the number of eggs per ovitrap were well below two and ten, respectively. Similarly, LF cases and high prevalence of microfilaria were reported from all study sites [17], [21] where *C. quinquefasciatus* was recorded in the present study except Dhunche located at more than 2,000 m asl. This may be due to a lower vector density or lack of disease diagnosis as this district is regarded as LF free. Moreover, LF is a chronic condition and recently infected asymptomatic cases may not have reported. Unfortunately, because of the short study period and lack of sufficiently disaggregated data, the temporal dynamics of DF and the association with its vectors could not be established in the present study. The reported DF cases in Nepal show a clear seasonal pattern [8], [83], [84] that can be related to temperature and rainfall with almost all cases in the monsoon and post-monsoon seasons. This pattern is consistent with reports from other countries in South-East Asia [4], [85]. Possible explanations for this seasonal transmission include vertical transmission of DENV in mosquitoes and, given the high number of asymptomatic DENV infections in the communities, silent transmission in people by a reduced number of vectors between the seasonal peaks [2]. A recent study suggests that a DENV-1 strain which is phylogenetically close to Indian viruses was responsible for the 2010 epidemic in Nepal and that this epidemic started in the southern lowland areas bordering India and then expanded to the mountain areas [84]. As DENV is a relatively recently introduced virus in Nepal and rapidly expanding its geographical range in the country, the implementation of both vertical (top-down) government-led and horizontal (bottom-up) community-led programmes is urgently required to limit its further spread and/or epidemic impact in the future.

Further studies that integrate serological, entomological, parasitological, socio-economic, climatic and environmental data along different altitudinal transects of Nepal appear urgently needed in the context of the rapid climate change that especially the higher altitudes of this country are experiencing. Moreover, there should be routine surveillance of DENV vectors and DF cases to prevent outbreaks. Similarly, xenomonitoring could be used for LF surveillance as the national programmes scales down MDA in known endemic areas, and to investigate the expansion of LF in Himalayan districts that were previously considered non-endemic.

### Conclusions and perspectives

The findings of our study may contribute to a better planning and scaling-up of mosquito-borne disease control programmes in the mountainous areas of Nepal that had previously been considered risk free. There is a higher preponderance and establishment of DENV vectors and DF cases up to the Middle Mountain region (1,310 m asl) supporting previous studies, while LF cases are reported up to 1,800 m asl and LF vectors up to 2,100 m asl in the High Mountain region. Climate change can increase the altitudinal ceiling of vector distribution and potentially put regions of Nepal that are presently sub-tropical and temperate at risk of DF and LF in the future provided that the domestic environments commonly exploited by the vectors are available in the climatically newly suitable areas. The knowledge of the vector dynamics and infection status of mosquitoes using xenomonitoring should be used for vector-borne disease surveillance in Nepal. In view of the high population density of mosquitoes in central Nepal, an integrated vector management programme based on control operations should be developed and implemented to particularly control the three mosquito species *A. aegypti*, *A. albopictus* and *C. quinquefasciatus*. We believe that xenomonitoring and control measures from the lowlands up to the High Mountain region are essential for the prevention of DF and LF in Nepal and therefore recommend that insecticide treatments or larval habitat perturbations should be conducted based on the surveillance of larvae in the areas at risk defined herein. The rapid range expansion of DENV vectors in a relatively short time up to the Middle Mountain region together with the presence of all four DENV serotypes and a large vulnerable human population in Nepal suggest that the risk of DF and its severe forms like DHF and DSS is much higher in this country than previously thought. Moreover, the abundance of LF vectors above 2,000 m in areas previously considered to be LF free challenges the government efforts to eliminate LF in Nepal by 2020. Therefore, urgent and responsible actions must be taken in coordination with related stakeholders to control DF and LF vectors in Nepal and their expansion into new areas. In addition to crucial community education and participation, cross-sectoral integration ensuring due consideration of the public health implications of transport management, urban development and planning, water supply, and waste and sewerage management are essential in this context.

## Supporting Information

Table S1 **Summary of the number of mosquitoes per month per study site.** BG-Sentinel trap (BGST) data is of two traps and CDC light trap (CDCLT) data of one trap per month per site.
(DOCX)

Table S2 **Regression model for predicting** ***Aedes aegypti*** **mean abundance using categorical explanatory variables.** Parameter estimates followed by the same letters are not statistically significant different from one another as revealed by Tukey's multiple comparisons. The p-values in bold print indicate significant differences.
(DOCX)

Table S3 **Regression model for predicting** ***Culex quinquefasciatus*** **mean abundance using categorical explanatory variables.** Parameter estimates followed by the same letters are not statistically significant different from one another as revealed by Tukey's multiple comparisons. The p-values in bold print indicate significant differences.
(DOCX)
