# Supplementary material for: Spatio-Temporal Distribution of Dengue and Lymphatic Filariasis Vectors along an Altitudinal Transect in Central Nepal
Source: PLoS Negl Trop Dis. 2014 Jul 31;8(7):e3035. doi: 10.1371/journal.pntd.0003035 (PMC4117448; doi:10.1371/journal.pntd.0003035)
Supplement: Table S1 — Summary of the number of mosquitoes per month per study site. BG-Sentinel trap (BGST) data is of two traps and CDC light trap (CDCLT) data of one trap per month per site. (DOCX) [file pntd.0003035.s001.docx]

| Table S1. Summary of the number of mosquitoes per month per study site | | | |  |  |  |  |  |
| --- | --- | --- | --- | --- | --- | --- | --- | --- |
| **Study sites and geographic coordinates** | **Trap method** | **Species** | **Year/Month** | | | | | |
|  |  |  | **2011** | | | | **2012** | |
|  |  |  | **September** | **October** | **November** | **December** | **January** | **February** |
| **Birgunj** |  |  |  |  |  |  |  |  |
| 26°59'59''N, 84°52'00''E | BGST | *Aedes aegypti* | 0 | 31 | 235 | 6 | 2 | 5 |
| 80 m above sea level (asl) | BGST | *Aedes albopictus* | 0 | 0 | 22 | 0 | 21 | 0 |
|  | BGST | *Culex quinquefasciatus* | 56 | 138 | 92 | 135 | 54 | 39 |
|  | CDCLT | *A*. *aegypti* | 0 | 0 | 5 | 1 | 0 | 0 |
|  | CDCLT | *A*. *albopictus* | 0 | 0 | 0 | 0 | 0 | 0 |
|  | CDCLT | *C*. *quinquefasciatus* | 106 | 49 | 333 | 31 | 76 | 0 |
| **Hetauda** |  |  |  |  |  |  |  |  |
| 27°25'02''N, 85°01'59''E | BGST | *A*. *aegypti* | 0 | 0 | 12 | 1 | 0 | 0 |
| 465 m asl | BGST | *A*. *albopictus* | 0 | 1 | 2 | 0 | 0 | 0 |
|  | BGST | *C*. *quinquefasciatus* | 9 | 28 | 32 | 18 | 8 | 35 |
|  | CDCLT | *A*. *aegypti* | 0 | 0 | 1 | 0 | 0 | 0 |
|  | CDCLT | *A*. *albopictus* | 0 | 0 | 2 | 0 | 0 | 3 |
|  | CDCLT | *C*. *quinquefasciatus* | 11 | 15 | 9 | 3 | 4 | 67 |
| **Kathmandu** |  |  |  |  |  |  |  |  |
| 27°41'59''N, 85°20'01''E | BGST | *A*. *aegypti* | 4 | 18 | 30 | 0 | 0 | 0 |
| 1,310 m asl | BGST | *A*. *albopictus* | 0 | 4 | 17 | 0 | 0 | 0 |
|  | BGST | *C*. *quinquefasciatus* | 0 | 26 | 32 | 0 | 0 | 0 |
|  | CDCLT | *A*. *aegypti* | 0 | 0 | 0 | 0 | 0 | 0 |
|  | CDCLT | *A*. *albopictus* | 5 | 0 | 0 | 0 | 0 | 0 |
|  | CDCLT | *C*. *quinquefasciatus* | 7 | 0 | 0 | 0 | 0 | 0 |
| **Ranipauwa** |  |  |  |  |  |  |  |  |
| 27°49'55''N, 85°14'21''E | BGST | *A*. *aegypti* | 0 | 0 | 0 | 0 | 0 | 0 |
| 1,825 m asl | BGST | *A*. *albopictus* | 0 | 0 | 0 | 0 | 0 | 0 |
|  | BGST | *C*. *quinquefasciatus* | 0 | 1 | 0 | 0 | 0 | 0 |
|  | CDCLT | *A*. *aegypti* | 0 | 0 | 0 | 0 | 0 | 0 |
|  | CDCLT | *A*. *albopictus* | 0 | 0 | 0 | 0 | 0 | 0 |
|  | CDCLT | *C*. *quinquefasciatus* | 0 | 1 | 0 | 0 | 0 | 0 |
| **Dhunche** |  |  |  |  |  |  |  |  |
| 28°06'45''N, 85°17'45''E | BGST | *A*. *aegypti* | 0 | 0 | 0 | 0 | 0 | 0 |
| 2,100 m asl | BGST | *A*. *albopictus* | 0 | 0 | 0 | 0 | 0 | 0 |
|  | BGST | *C*. *quinquefasciatus* | 2 | 10 | 0 | 0 | 0 | 0 |
|  | CDCLT | *A*. *aegypti* | 0 | 0 | 0 | 0 | 0 | 0 |
|  | CDCLT | *A*. *albopictus* | 0 | 0 | 0 | 0 | 0 | 0 |
|  | CDCLT | *C*. *quinquefasciatus* | 4 | 0 | 0 | 0 | 0 | 0 |
