# Supplementary material for: Spatio-Temporal Distribution of Dengue and Lymphatic Filariasis Vectors along an Altitudinal Transect in Central Nepal
Source: PLoS Negl Trop Dis. 2014 Jul 31;8(7):e3035. doi: 10.1371/journal.pntd.0003035 (PMC4117448; doi:10.1371/journal.pntd.0003035)
Supplement: Table S3 — Regression model for predicting Culex quinquefasciatus mean abundance using categorical explanatory variables. Parameter estimates followed by the same letters are not statistically significant different from one another as revealed by Tukey's multiple comparisons. The p-values in bold print indicate significant differences. (DOCX) [file pntd.0003035.s003.docx]

**Table S3.** Regression model for predicting *Culex quinquefasciatus* mean abundance using categorical explanatory variables.

|  |  | | |  |  |  |  |  |  |
| --- | --- | --- | --- | --- | --- | --- | --- | --- | --- |
| **Parameters** | | | **Regression coefficients** | | | **Std. Error** | | **z value** | **Pr(>\|z\|)** |
| (Intercept) | | 4.461 | | | | 0.695 | | 6.421 | **<0.001** |
| Region: Siwalik^a^ | | -1.485 | | | | 0.666 | | -2.230 | **0.026** |
| Region: Middle Mountain^b^ | | -3.686 | | | | 0.695 | | -5.304 | **<0.001** |
| Region: High Mountain^c^ | | -5.538 | | | | 0.680 | | -8.142 | **<0.001** |
| Month: Oct11^a^ | | 0.808 | | | | 0.775 | | 1.043 | 0.297 |
| Month: Nov11 | | 0.315 | | | | 0.785 | | 0.402 | 0.688 |
| Month: Dec11 | | -1.437 | | | | 0.856 | | -1.679 | 0.093 |
| Month: Jan12^b^ | | -2.159 | | | | 0.900 | | -2.398 | **0.016** |
| Month: Feb12 | | -0.799 | | | | 0.846 | | -0.945 | 0.345 |
